# HO-1 drives autophagy as a mechanism of resistance against HER2-targeted therapies

**DOI:** 10.1007/s10549-019-05489-1

**Published:** 2019-11-08

**Authors:** Natasha Tracey, Helen Creedon, Alain J. Kemp, Jayne Culley, Morwenna Muir, Teresa Klinowska, Valerie G. Brunton

**Affiliations:** 1 Edinburgh Cancer Research UK Centre, Institute of Genetics & Molecular Medicine, University of Edinburgh, Crewe Road South, Edinburgh, EH4 2XR UK; 2 Oncology, IMED Biotech Unit, AstraZeneca, Cambridge, UK

**Keywords:** HER2, Breast cancer, HO-1, Autophagy, Resistance

## Abstract

**Purpose:**

Targeted therapies have resulted in major advances in the treatment of HER2-positive breast cancers. Despite this, up to 70% of patients will develop resistance to treatment within 2 years and new strategies for targeting resistant disease are needed.

**Methods:**

To identify potential resistance mechanisms, we used the mouse MMTV-NIC-PTEN^+/−^ spontaneous model of HER2-positive breast cancer and the pan-HER family kinase inhibitor sapatinib. Vehicle and sapatinib-treated tumors were evaluated by immunohistochemistry and proteomic analysis. In vitro studies were carried out to define the role of heme oxygenase 1 (HO-1) and autophagy in resistance to sapatinib and lapatinib, another pan-HER family kinase inhibitor.

**Results:**

Treatment of tumor-bearing MMTV-NIC-PTEN^+/−^ mice with sapatinib resulted in delayed tumor progression and increased survival. However, tumors eventually progressed on treatment. Proteomic analysis identified proteins associated with cellular iron homeostasis as being upregulated in the sapatinib-treated tumors. This included HO-1 whose overexpression was confirmed by immunohistochemistry. Overexpression of HO-1 in HER2-expressing SKBR3 breast cancer cells resulted in reduced sensitivity to both pan-HER family kinase inhibitors sapatinib and lapatinib. This was associated with increased autophagy in the HO-1 over-expressing cells. Furthermore, increased autophagy was also seen in the sapatinib-treated tumors. Treatment with autophagy inhibitors was able to increase the sensitivity of the HO-1 over-expressing cells to both lapatinib and sapatinib.

**Conclusion:**

Together these data indicate a role for HO-1-induced autophagy in resistance to pan-HER family kinase inhibitors.

**Electronic supplementary material:**

The online version of this article (10.1007/s10549-019-05489-1) contains supplementary material, which is available to authorized users.

## Introduction

HER2 is a member of the human epidermal growth factor receptor (EGFR) family which consists of four members (HER1, HER2, HER3 and HER4). It is overexpressed in approximately 15–20% of breast cancers where it is associated with poor prognosis [1]. A number of HER2-targeted therapies have been developed, the first of which was the monoclonal antibody trastuzumab [2]. In combination with chemotherapy, trastuzumab is currently first-line treatment for patients with HER2-positive breast cancer. Other drugs targeting HER2 have subsequently been developed, including the monoclonal antibody pertuzumab and the small molecule tyrosine kinase inhibitors lapatinib, sapatinib and neratinib [3–6].

Although the introduction of HER2-targeted therapies has had a major impact on the treatment of the disease, resistance remains a significant clinical problem. Both de novo and acquired resistance impact detrimentally on patient outcomes, reducing progression-free survival. Several mechanisms of resistance have been identified in preclinical models, but these have proven difficult to translate into clinical benefit [7–9]. This is in part due to the complexity and heterogeneity of the disease which is often not captured in preclinical models using established cell lines [10]. One alternative approach is to use genetically engineered mouse models which allow autochthonous tumor growth in immune-competent hosts [11].

For this reason, we have exploited the genetically engineered MMTV-NIC (Neu-IRES-Cre) mouse model of HER2-driven mammary tumorigenesis [12]. In this model, HER2 expression is driven by MMTV-Cre in the mammary epithelium using a bicistronic transcript to co-express activated ErbB2/Neu (HER2) with MMTV-Cre recombinase. Using this approach, we have previously demonstrated that genetic loss of phosphatase and tensin homologue (PTEN) in HER2-driven mammary tumors confers resistance to the tyrosine kinase inhibitor sapatinib [13]. Sapatinib treatment resulted in tumor shrinkage in the majority of MMTV-NIC-PTEN^+/+^ mice, but despite slowing tumor growth in MMTV-NIC-PTEN^+/−^ mice, it did not cause tumor resolution.

Using a proteomic approach, we identified heme oxygenase 1 (HO-1) as being significantly upregulated in sapatinib-treated tumors from MMTV-NIC-PTEN^+/−^ mice. HO-1 is the rate limiting enzyme in the breakdown of heme groups into biliverdin, releasing carbon monoxide and iron in the process. HO-1 is also induced in response to a number of cellular stresses in pathological conditions where it exerts strong antioxidant and anti-inflammatory functions. As such, modulation of HO-1 expression has emerged as a potential therapeutic target for certain cardiovascular and neurodegenerative diseases where it provides a cytoprotective function [14]. In contrast, in the context of cancer HO-1 overexpression has been reported in a number of tumor types, including breast, where it is associated with poor prognosis [15, 16]. Overexpression of HO-1 in experimental models has been shown to increase proliferation and promote survival of cancer cells and tumor growth in vivo although opposing effects have been reported suggesting tumor type specific effects [15, 16]. In addition, HO-1 expression is also induced in response to chemo- and radiation therapy, and has been implicated in both drug- and therapy-induced resistance [17–19].

Autophagy is a catabolic process that is activated in response to cellular stress that allows the cell to degrade intracellular aggregated or misfolded proteins and damaged organelles. Deregulation of autophagy in cancer can have both pro- and anti-survival roles and is determined by nutrient availability, microenvironmental stress and immune signals [20]. A similar paradoxical role for autophagy in response to therapy has been reported where induction of autophagy can result in either autophagic cell death or be activated as a protective mechanism that mediates acquired resistance to therapy [21]. Here we show that autophagy is induced in sapatinib-treated tumors in MMTV-NIC-PTEN^+/−^ mice and that ectopic expression of HO-1 in the human HER2-overexpressing cell line, SKBR3, reduces sensitivity to both sapatinib and lapatinib, and confers resistance in an autophagy-dependent manner.

## Materials and methods

### Mice

MMTV-NIC-PTEN^+/−^ mice were generated as previously described [13]. All experiments were conducted in compliance with UK Home Office guidelines. Nulliparous females were monitored twice weekly, using manual palpation, for tumor formation. The greatest tumor dimension and its perpendicular measurement were recorded, and when tumors had reached their maximal size (15 mm in one direction) as determined by Home Office regulations, mice were sacrificed. Tumors were then collected and fixed in 10% neutral buffered formalin or flash frozen in liquid nitrogen. For drug studies with sapatinib (AZD8931, AstraZeneca, UK), treatment commenced when a tumor reached ≥ 0.1 cm^3^ and continued until mice were sacrificed due to tumor burden. Mice were dosed once daily by oral gavage with vehicle (1% Tween80 in PBS) or sapatinib (100 mg/kg in 1% Tween80 in PBS).

### Immunohistochemistry

Immunohistochemistry of formalin-fixed, paraffin-embedded tissues was performed as described previously [22]. Primary antibodies used were as follows: PTEN (catalogue no. 9188; 1:200; Cell Signaling, MA, USA), phospho-Akt (Ser 473; catalogue no. 4060; 1:50; Cell Signaling, MA, USA), pErk1/2 (Thr202/Tyr204; catalogue no. 4370; 1:250; Cell Signaling, MA, USA), HO-1 (catalogue no. ab13248; 1:250; Abcam, Cambridge, UK), NRF2 (catalogue no. ab31163; 1:100; Abcam, Cambridge, UK), LC3-B (catalogue no. 0231-100; 1:100; Nanotools, Germany), p62 (catalogue no. BMLPW9860; 1:1000; Enzo Life Sciences, NY, USA). Staining was scored using Definiens Architect Tissue Studio software (Definiens, Germany) and statistical analysis, comparing percentage of positively stained cells, performed using Prism (GraphPad, CA, USA).

### Proteomics

Tumors from MMTV-NIC-PTEN^+/−^ mice were homogenized in 8 M urea, 50 mM ammonium bicarbonate with protease and phosphatase inhibitors. Samples were denatured with 8 mM dithiothreitol (10 min; 50 °C) and alkylated with 16 mM iodoacetamide (10 min; 37 °C). Samples were digested with trypsin overnight at 37 °C (1:200 enzyme:protein). Peptide extracts were then cleaned on stage tips before being analysed on a Thermo RSLC 3000 Nano with a home-packed 10 cm × 75 μm, 1.8 μm C18 particle, home-pulled packed emitter, coupled to Q-Exactive Plus instrument (Thermo Fisher Scientific, MA, USA). 15 min loading at 0.4 μl/min, 2% organic (acetonitrile; 0.05% acetic acid) was followed by a 120 min linear gradient to 50% organic, and 80% wash; 2% equilibration completed the method. MS1 scan range was 300–1650, AGC 3 × 10^6^ and max ion time 60 ms. Data were processed in MaxQuant version 1.5.6.5 [23] with Uniprot mouse reference proteome and normalized LFQ was generated with match-between-runs enabled.

### Cell culture

SKBR3 and MDA-MB-231 human breast cancer cell lines were purchased from the American Type Culture Collection and grown in Dulbecco’s Modified Eagle’s medium supplemented with 2 mM l-glutamine and 10% FBS. Cells were maintained at 37 °C in a humidified atmosphere with 5% CO_2_. To generate stable cell lines, SKBR3 cells were nucleofected with 1 μg pCMV6-AC-GFP or pCMV6-AC-GFP-HMOX1 (RG200463; Origene, MD, USA). Cells containing the appropriate plasmid were selected using 600 μg/ml G418 for 3 weeks, then FACS sorted.

### AlamarBlue assay

Cells were seeded in 96-well plates and allowed to attach for 24 h. Escalating doses of lapatinib (GlaxoSmithKline, UK) or sapatinib (AZD8931; AstraZeneca, UK) prepared in DMSO were then added. After 72 h, AlamarBlue cell viability reagent (ThermoFisher Scientific, MA, USA) was added and fluorescence measured after a further 60 min. Mean values were calculated from six replicate wells and normalized against the mean value of the vehicle-treated wells. GI50 values were generated using Prism (GraphPad, CA, USA) from a minimum of four biological repeats.

### Cell death assay

Cells were seeded in 12-well plates and allowed to attach for 24 h. Lapatinib (5 μM in DMSO; GlaxoSmithKline, UK), sapatinib (0.67 μM in DMSO; AstraZeneca, UK), 3-methyladenine (5 mM in water; Sigma Aldrich, MO, USA) or bafilomycin A1 (5 nM in DMSO; Sigma Aldrich, MO, USA) were added to cells for 48 h. After this, cells were removed from dishes and 1 μg/ml propidium iodide (Sigma Aldrich; MO, USA) added for 5 min. Cell death was read on a Tali Image-based Cytometer (Invitrogen, CA, USA) or Accuri™ C6 Flow Cytometer (BD Biosciences). All conditions were compared to DMSO control and single agent treatments. Statistical analysis was performed using Prism (GraphPad, CA, USA) from a minimum of three biological repeats.

### Glucose 6 phosphate dehydrogenase (G6PD) assay

G6PD activity was measured in tissue homogenates in PBS using a G6PD activity assay kit based on the oxidation of glucose 6 phosphate to gluconolactone by G6PD, as per the manufacturers protocol (Abcam, Cambridge). Samples were measured in duplicate over 30 min and kinetic curves generated.

### Glutathione assay

Reduced and oxidized glutathione concentration was determined in tissue homogenates using a GSH/GSSG ratio detection assay kit based on the detection of a fluorescent thiol green dye following reaction with GSH, as per the manufacturers protocol (Abcam, Cambridge, UK). Samples were measured in duplicate.

### Western blotting

Tumors were homogenized using the FastPrep-24 homogeniser (MP Biomedicals, CA, USA) and lysed in 1% (v/v) Triton X-100, 50 mM HEPES (pH 7.4) 150 mM NaCl, 1.5 mM MgCl_2_, 1 mM EGTA, 100 mM NaF, 10 mM Na_4_P_2_O_7_, 1 mM Na_3_VO_4_, 10% (v/v) glycerol, cOmplete^TM^, EDTA-free Protease Inhibitor Cocktail and PhosSTOP^TM^ (Roche) at 4 °C. Cells were washed twice in ice cold PBS and lysed in RIPA buffer (50 mM Tris pH 8.0, 150 mM NaCl, 1% TritonX-100, 0.5% sodium deoxycholate, 0.1% SDS) containing cOmplete^TM^, EDTA-free Protease Inhibitor Cocktail and PhosSTOP^TM^ (Roche) at 4 °C prior to SDS-PAGE. Proteins were transferred to nitrocellulose and membranes incubated with antibodies to HO-1 (catalogue number 5853; 1:1000), p62 (catalogue number 5114; 1:1000), pErk1/2 (Thr202/Tyr204) (catalogue number 4370; 1:1000), Erk1/2 (catalogue number 4696; 1:1000), pJNK (Thr183/Tyr185) (catalogue number 9251; 1:1000), JNK (catalogue number 9252; 1:1000) (all Cell Signaling, MA, USA) or LC3B (catalogue number L7543; 1:3000; Sigma Aldrich, MO, USA) overnight at 4 °C. Membranes were washed thrice with TBST before incubation with HRP-conjugated secondary antibodies (Cell Signaling, MA, USA1:1000). Washes were repeated in TBST and bound antibodies detected by chemiluminescence using Clarity ECL Substrate (BioRad, Germany) on the BioRad Gel Dox XR+.

## Results

### Sapatinib delays tumor progression

**Fig. 1:**
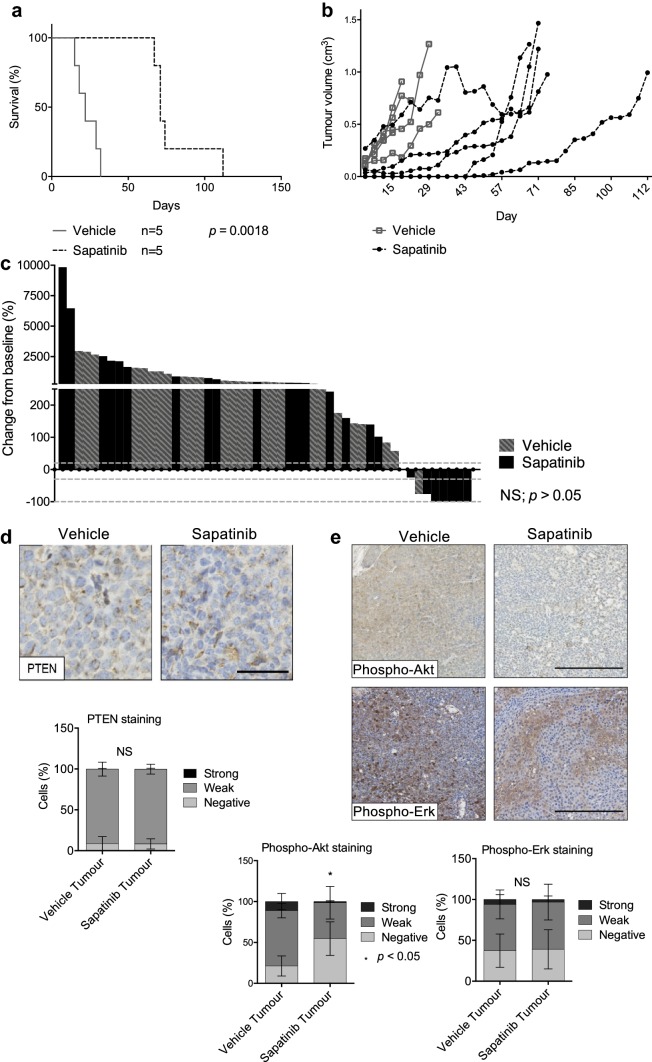
Sapatinib increases survival in MMTV-NIC-PTEN^+/−^ mice. **a** Kaplan–Meier survival curve showing survival time from start of treatment in MMTV-NIC PTEN^+/−^ mice treated with vehicle or sapatinib. Log-rank test, *p *= 0.0018. **b** Graph showing growth curves of index tumors in MMTV-NIC-PTEN^+/−^ mice treated with vehicle or sapatinib from the onset of the first palpable tumor. **c** Waterfall plot showing volume change from baseline (%) of all MMTV-NIC-PTEN^+/−^ tumors from mice treated with vehicle or sapatinib. Dotted lines represent 20% increase, 30% decrease and 100% decrease in tumor volume from baseline, respectively. Two-tailed Mann–Whitney test, *p *= 0.0682. **d** Representative immunohistochemical staining of PTEN (vehicle: *n *= 24; sapatinib: *n *= 12). Scale bar 50 μm. Quantification of PTEN staining by Definiens Architect (bottom panel). Results presented as mean ± standard deviation. Two-tailed Mann–Whitney test, not significant = NS. **e** Representative immunohistochemical staining of phospho-Akt (vehicle: *n *= 11; sapatinib: *n *= 6) and phospho-Erk (vehicle: *n *= 25; sapatinib: *n *= 10). Scale bar: 250 µm. Quantification of phospho-Akt and phospho-Erk IHC staining by Definiens Architect (bottom panels). Results presented as mean ± standard deviation. Two-tailed Mann–Whitney test, not significant = NS, *p *< 0.05*

Tumor-bearing MMTV-NIC-PTEN^+/−^ mice were randomised to receive vehicle (1% Tween80 in PBS; po, q.d.) or sapatinib (100 mg/kg, with 1% Tween80 in PBS; po, q.d.). Vehicle-treated mice had a median survival time of 22 days after onset of treatment, compared to 71 days for sapatinib-treated mice (Fig. 1a; HR: 0.3099; CI of ratio: 0.08970–1.070; *p *= 0.0018). MMTV-NIC-PTEN^+/−^ mice can develop multiple mammary tumors: mice treated with vehicle developed a median of 6 tumors each, whereas mice on sapatinib treatment developed a median of 5 tumors each (Mann–Whitney test; CI (−3)–4; *p *= 0.175; NS).

The index tumor was defined as the largest tumor at the time of sacrifice. Growth kinetics of the index tumors from each mouse (Fig. 1b) showed that tumors from vehicle-treated mice grew rapidly after the initial tumor onset, whereas index tumors from sapatinib-treated mice had a longer period of latency after treatment commenced before rapid growth began. These index tumors represent tumors that have progressive disease in the continued presence of sapatinib, providing a good model with which to study mechanisms of resistance that models continued adjuvant treatment with HER2 targeted therapies given to patients.

Using RECIST guidelines [24], 83% of all tumors from both treatment arms combined were classed as progressive disease having greater than 20% increase in tumor volume (Fig. 1b; vehicle: *n *= 25 [92.59%]; sapatinib: *n *= 17 [70.83%]). Two tumors were between 20% increase and 30% decrease in tumor volume (Fig. 1c; vehicle: *n *= 0 [0%]; sapatinib: *n *= 2 [8.34%]) and were thus classified as stable. Several tumors that became palpable after treatment commenced showed pathological complete response (pCR) to treatment, with no palpable tumor detected (Fig. 1c; vehicle: *n *= 0 [0%]; sapatinib: *n *= 5 [20.84%]). Two tumors had partial responses, greater than 30% regression, but less than 100% regression (Fig. 1c; vehicle: *n *= 1 [3.70%]; sapatinib: *n *= 1 [4.17%]). No significant difference was seen between treatment groups when comparing the overall percentage volume change from baseline (Mann–Whitney test; *p *= 0.0682; NS).

Previous work has reported that loss of PTEN can confer resistance to trastuzumab and sapatinib [13, 25, 26]. However, analysis of PTEN in the tumors from the MMTV-NIC-PTEN^+/−^ mice showed that the resistant tumors did not result from the outgrowth of a PTEN-deficient population of cells following sapatinib treatment (Fig. 1d). The PI3 K/Akt and MAPK pathways are downstream of HER2 signaling. Inhibition of HER2 by sapatinib has been shown to reduce signaling through these pathways in MMTV-NIC-PTEN^+/−^ mice after 3 days of treatment with sapatinib [13]. To test if PI3 K/Akt and MAPK pathways remained inhibited in the tumors that were able to grow through sapatinib treatment, immunohistochemistry was carried out for phospho-Akt and phospho-Erk. Levels of phospho-Akt were significantly reduced in sapatinib-treated tumors (Fig. 1e; median [CI]: vehicle: 80.18% (64.37–87.20); sapatinib: 36.45% (31.29–80.83); *p *< 0.05), although no significant difference could be seen between treatment groups when comparing levels of phospho-Erk (Fig. 1e; median [CI]: vehicle: 63.75% [54.93–73.13]; sapatinib: 63.22% [35.01–89.43]; NS). However, expression of phospho-Erk was highly variable across the tumors whereas phospho-Akt expression was more consistent across the whole tumor section (Fig. 1e).

### Proteomic analysis reveals changes in iron homeostasis in sapatinib-treated tumors

**Fig. 2:**
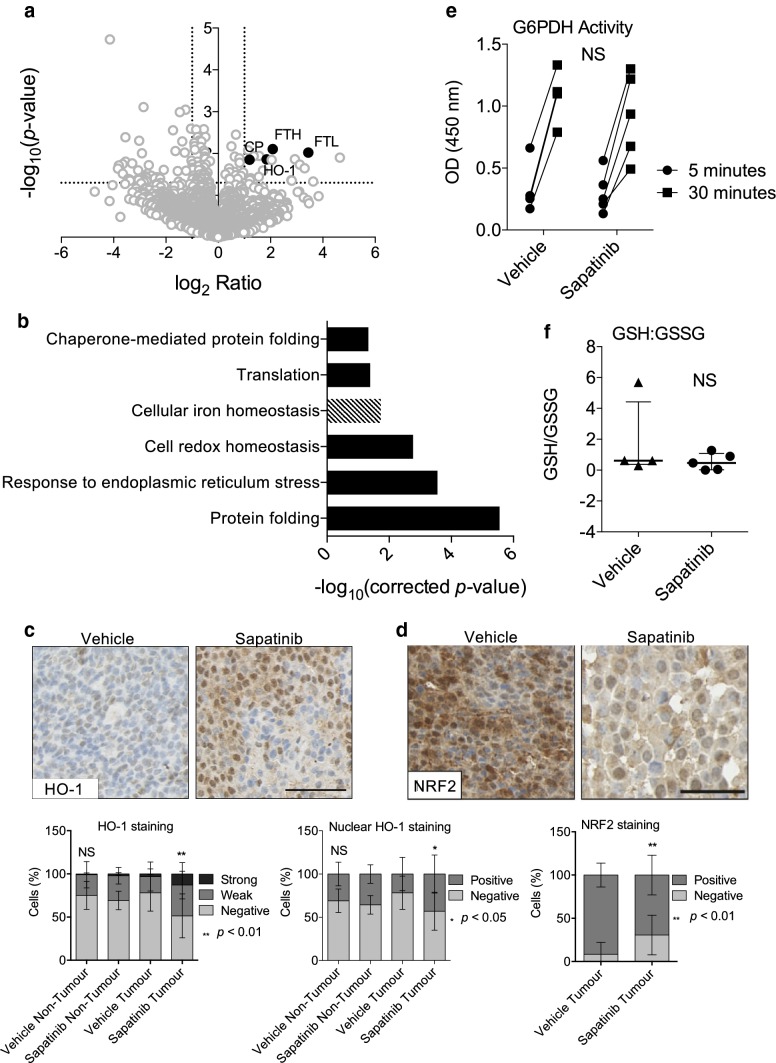
Proteomic analysis reveals alterations in cellular iron homeostasis in sapatinib-treated tumors. Index tumors from MMTV-NIC-PTEN^+/−^ mice treated with vehicle (*n *= 4) or sapatinib (*n *= 5) were subject to liquid chromatography-tandem mass spectrometry (LC–MS/MS). Protein identification and quantitation were performed using MaxQuant. **a** Volcano plot of proteins identified by MaxQuant. Highlighted are proteins associated with cellular iron homeostasis, ferritin heavy chain (FTH), ferritin light chain (FTL), ceruloplasmin (CP) and heme oxygenase-1 (HO-1). Dotted lines represent *p *= 0.05 and twofold ratio. **b** Bar chart showing significantly upregulated gene ontology (GO) terms related to proteins that were differentially regulated between vehicle-treated and sapatinib-treated tumors. Dashed bars represent GO terms upregulated in sapatinib-treated tumors, whereas solid bars represent those downregulated in sapatinib-treated tumors. Differentially regulated genes were selected as those which showed > twofold change and *p *< 0.05, as determined by MaxQuant, Wilcoxon–Mann–Whitney test. Benjamini–Hochberg corrected *p*-values and GO terms obtained from DAVID. **c** Representative immunohistochemical staining of HO-1 (vehicle: *n *= 23; sapatinib: *n *= 9). Scale bar: 50 µm. Quantification of immunohistochemical staining by Definiens Architect (bottom panels). Results presented as mean ± standard deviation. Kruskal–Wallis test, Dunn’s post hoc test, not significant = NS, *p *< 0.05*, *p *< 0.01**. **d** Representative immunohistochemical staining of NRF2 (vehicle: *n *= 20; sapatinib: *n *= 9). Scale bar: 50 µm. Quantification of immunohistochemical staining by Definiens Archtect (bottom panel). Results presented as mean ± standard deviation. Two-tailed Mann–Whitney test*, *p *< 0.01**. **e** G6PDH kinetic assay with measurements at 5 and 30 min in vehicle (*n *= 4) and sapatinib-treated (*n *= 5) tumors. Linear regression, not significant = NS. **f** GSH/GSSG ratio determined in vehicle (*n *= 4) and sapatinib-treated (*n *= 5) tumors. Results presented as scatter plot with median ±interquartile range. Two-tailed Mann–Whitney test, not significant = NS

To assess differentially regulated pathways in sapatinib-treated tumors compared to vehicle-treated tumors, index tumors from both treatment arms were subjected to proteomic analysis. This revealed a small number of proteins that were significantly (*p *< 0.05) and twofold differentially regulated between vehicle-treated and sapatinib-treated tumors (*n *= 123, increased: *n *= 34, decreased: *n *= 89; Fig. 2a). Gene ontology (GO) analysis revealed several proteins upregulated in sapatinib-treated tumors related to control of cellular iron homeostasis (*n *= 4; Fig. 2b; *p *= 0.019). In addition, reduced expression of proteins involved in cellular redox homeostasis (*n *= 6; *p *= 0.0017), response to endoplasmic reticulum stress (*n *= 7; *p *= 0.00028) and protein folding (*n* = 10; *p *< 0.00001) were identified in sapatinib-treated tumors compared to vehicle-treated tumors (Fig. 2b).

As cellular iron homeostasis was the only GO term upregulated in sapatinib-treated tumors compared to vehicle-treated tumors, it was decided that this represented a good avenue to investigate. One protein identified and confirmed as increased in sapatinib-treated tumors was HO-1 (Fig. 2c; median [CI]: non-tumor: vehicle: 23.06% [13.76–32.12]; sapatinib: 27.14% [21.99–46.82]; NS; tumor: vehicle: 14.31% [8.882–26.17]; sapatinib: 50.61% [23.81–73.39]; *p *< 0.01). Significantly higher levels of nuclear HO-1 were also seen in sapatinib-treated tumors (Fig. 2c). HO-1 has previously been linked to resistance to targeted- and chemo-therapies in a number of cancer types [27]. Due to this, we focused on the role of HO-1 in response to sapatinib treatment.

HO-1 is linked to the oxidative stress response and is a key effector of nuclear factor erythroid 2-related factor 2 (NRF2)-mediated maintenance of redox homeostasis [28]. However, levels of NRF2 were lower in the sapatinib-treated tumors (Fig. 2d; median [CI]: vehicle: 98.05% [86.40–99.50]; sapatinib: 71.36% [46.39–92.96]; *p *< 0.01). Cellular oxidative stress can be measured by looking at the level of nicotinamide adenine dinucleotide phosphate (NADPH) available to provide reducing power. To measure this, we looked at the activity of glucose-6-phosphate dehydrogenase (G6PDH) which catalyzes the rate limiting step in the pentose phosphate pathway, responsible for maintaining cellular levels NADPH [29]. Both sapatinib-treated and vehicle-treated tumors showed similar levels of G6PDH activity (Fig. 2e; slope [CI]: vehicle: 0.03973 [0.01444–0.04502]; sapatinib: 0.02483 [0.00901–0.04066]; NS). Reduced glutathione (GSH) is another key cellular antioxidant; increased oxidized glutathione (GSSG) is indicative of oxidative stress [30]. The ratio of GSH to GSSG was not significantly different between vehicle-treated and sapatinib-treated tumors (Fig. 2f; median [CI]: vehicle: 0.6186 [0.2980–5.689]; sapatinib: 0.4666 [0.0009–1.2710]). The increased HO-1 in the sapatinib-treated tumors is therefore not linked to increased activity of an NRF-2 mediated oxidative stress response.

### Induction of autophagy as a mechanism of resistance

**Fig. 3:**
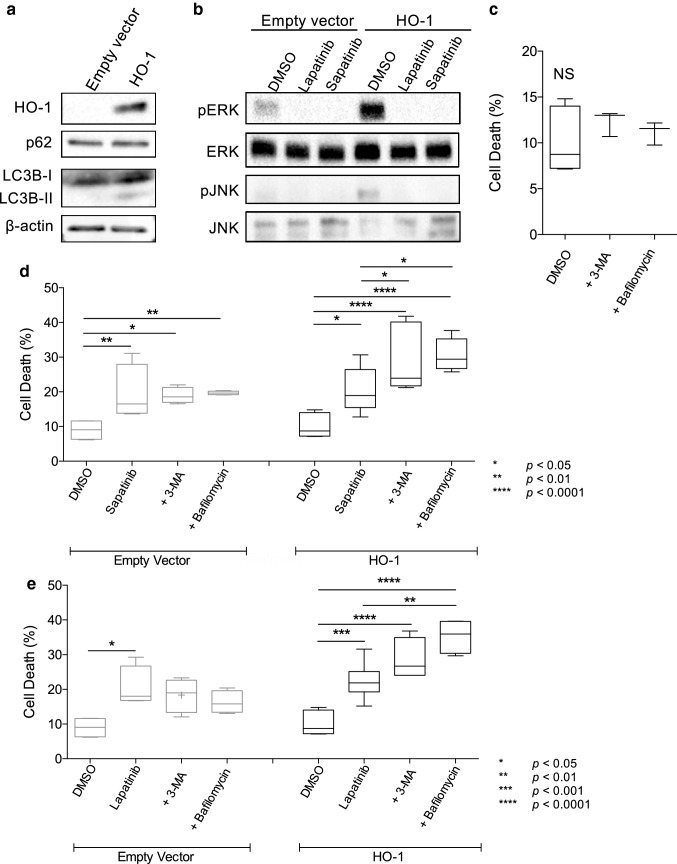
Increased autophagy as a mechanism of resistance. **a** Western blot analysis of HO-1, p62 and LC3B expression in SKBR3 breast cancer cell lines stably transfected with a plasmid containing the heme oxygenase-1 (HO-1) gene or an empty plasmid. β-actin was used as a loading control. **b** Western blot analysis of SKBR3 empty vector and HO-1 expressing cells following treatment with DMSO, lapatinib or sapatinib for 2 h. **c**–**e** Cells were treated for 48 h with **c** dimethyl sulfoxide (DMSO; 0.01%), **d** sapatinib (0.67 µM) or **e** lapatinib (5 µM) in the presence or absence of autophagy inhibitors 3-methyladenine (3-MA; 5 mM) or bafilomycin A1 (bafilomycin; 5 nM). Cells were stained with propidium iodide and percentage of cell death was analysed using a Tali Image Cytometer. Results presented as box and whisker plot, minimum of three biological repeats. All conditions were compared to DMSO control and single agent treatments. One-way ANOVA, Bonferroni’s post hoc test, not significant = NS, *p *< 0.05*, *p *< 0.01**, *p *< 0.001***

As HO-1 was increased in sapatinib-treated tumors and it has been reported to protect cells against a number of chemotherapeutic agents we asked whether HO-1 could protect cells from lapatinib and sapatinib. HO-1 was overexpressed in the human HER2 overexpressing breast cancer cell line SKBR3 (Fig. 3a). This resulted in a fivefold and fourfold increase in GI50 values for sapatinib and lapatinib respectively (Table 1).

**Table 1:** GI50 values for sapatinib and lapatinib in SKBR3 and SKBR3-HO-1 cells

Cell line	Drug	
	Sapatinib (M)^a^	Lapatinib (M)^a^
SKBR3	5.83 × 10^−12^	1.12 × 10^−12^
SKBR3 HO-1	9.53 × 10^−7^	4.31 × 10^−8^

^a^GI50 values

HO-1 has been shown to increase autophagic flux as a resistance mechanism in a number of tumor types [31–34]. Induction of autophagy in HO-1 overexpressing cells, was evidenced by an increased ratio of soluble microtubule-associated protein light chain (LC3BI) to membrane bound LC3BII which marks the formation of autophagosomes (Fig. 3a). However, levels of sequestosome 1 (p62), which targets ubiquitinated proteins for autophagic degradation, were similar between empty vector and HO-1 overexpressing cells (Fig. 3a). Furthermore, there was increased activation of ERK and JNK, both of which have been associated with autophagy regulation, in the HO-1 overexpressing cells (Fig. 3b).

To address whether induction of autophagy could protect cells from the effects of sapatinib and lapatinib, we treated cells with the autophagy inhibitors 3-methyladenine (3-MA) or bafilomycin A1. Treatment with either 3-MA or bafilomycin A had no effect on cell death (Fig. 3b; mean cell death% [SD]: DMSO: 10.41 [± 3.53]). Additionally, co-treatment of empty vector cells with sapatinib or lapatinib and 3-MA or bafilomycin A1 resulted in little change in cell death (Fig. 3c, d; mean cell death% [SD]: DMSO: 8.99 [± 3.00]; sapatinib: 19.44 [± 8.06]; sapatinib + 3-MA: 18.92 [± 2.29]; sapatinib + bafilomycin: 19.68 [± 0.52]; lapatinib: 20.52 [± 5.92]; lapatinib + 3-MA: 18.34 [± 4.86]; lapatinib + bafilomycin: 16.28 [3 ± 0.26]). However, in HO-1 overexpressing cells autophagy inhibition significantly increased cell death in response to both sapatinib and lapatinib (Fig. 3c, d; mean cell death% [SD]: DMSO: 10.41 [± 3.53]; sapatinib: 20.01 [± 5.97]; sapatinib + 3-MA: 29.54 [± 9.80]; sapatinib + bafilomycin: 30.69 [± 4.70]; lapatinib: 22.19 [± 4.70]; lapatinib + 3-MA: 28.57 [± 6.03]; lapatinib + bafilomycin: 35.31 [± 5.04]). Thus, in the context of HO-1 overexpression, autophagy is an important mechanism of resistance to saptinib and lapatinib-induced cell death. Co-treatment of MDA-MB-231 cells, which do not express HER2 but express EGFR, with sapatinib or lapatinib and 3-MA or bafilomycin significantly increased cell death (Supplementary Fig. 1a). Western blotting showed that HO-1 was not detectable in the MDA-MB-231 cells (Supplementary Fig. 1b). Thus, autophagy also plays a role in the sensitivity to both lapatinib and sapatinib in non-HER2-driven cells although this does not appear to be dependent on HO-1.

**Fig. 4:**
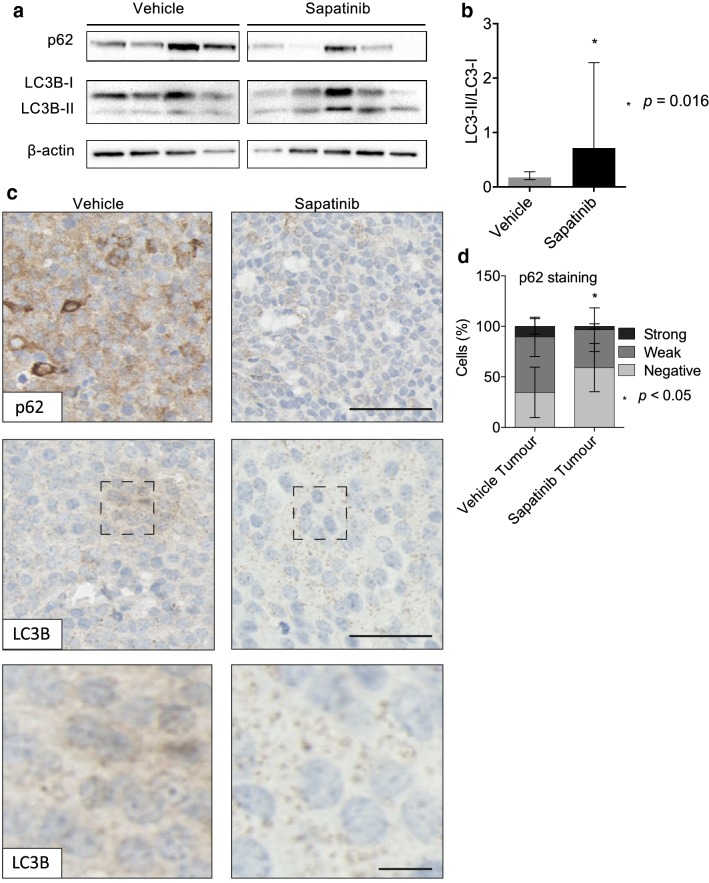
Increased autophagy in tumors progressing on sapatinib. **a** Western blot analysis of p62 and LC3B in index tumors from MMTV-NIC-PTEN^+/−^ mice treated with vehicle (*n *= 4), or sapatinib (*n *= 5). β-actin was used as a loading control. **b** Quantification of LC3BI:LC3BII. Results presented as bar graph, mean ± standard deviation (SD). Two-tailed Mann–Whitney test, *p *= 0.016. **c** Representative immunohistochemical staining of p62 (vehicle: *n *= 22; sapatinib: *n *= 10) and LC3B (vehicle: *n *= 19; sapatinib: *n *= 8). Scale bar: 50 µm. Higher magnification of LC3B puncta. Scale bar: 10 µm. **d** Quantification of p62 by Definiens Architect. Results presented as mean ± SD, two-tailed Mann–Whitney test, *p *< 0.05*

We then asked whether there was evidence of increased autophagy in the sapatinib-treated tumors. Levels of the autophagy-related proteins p62 and LC3B were assessed. Western blot analysis revealed decreased p62 in sapatinib-treated tumors (Fig. 4a) and increased autophagic flux, evidenced by an increased ratio of LC3BI to LC3BII (Fig. 4a, b). These results were mirrored by immunohistochemical analysis. Sapatinib-treated tumors had lower levels of p62 than vehicle-treated tumors (Fig. 4c, d). In addition, very few LC3 puncta were seen in tumors from vehicle-treated mice, but this was increased in the tumors that were progressing on sapatinib (Fig. 4d). Therefore, HO-1 expression correlates with autophagy induction in response to sapatinib in vivo.

## Discussion

Using a HER2-driven genetically engineered mouse model has allowed us to identify a novel mechanism of resistance to HER2-targeted therapies. In this model, the initial response to sapatinib treatment was followed by a period of rapid disease progression. Analysis of tumors which were progressing while on treatment revealed a significant increase in HO-1. The primary role for HO-1 is in the breakdown of heme groups into biliverdin, releasing carbon monoxide and iron in the process. In addition to this homeostatic role, HO-1 is also induced in response to a number of cellular stresses in pathological conditions where it exerts strong antioxidant and anti-inflammatory functions. As such, modulation of HO-1 expression has emerged as a potential therapeutic target for certain cardiovascular and neurodegenerative diseases where it provides a cytoprotective function [14].

In contrast, in the context of cancer HO-1 overexpression has been reported in a number of tumor types, including breast, where it is associated with poor prognosis [15, 16]. Overexpression of HO-1 in experimental models has been shown to increase proliferation and promote survival of cancer cells and tumor growth in vivo although opposing effects have been reported suggesting tumor type specific effects [15, 16]. In addition, HO-1 has been implicated in drug- and therapy-induced resistance [17–19]. Consistent with these observations, our data indicate a role for HO-1 in resistance to sapatinib and lapatinib with HO-1 overexpressing SKBR3 cells displaying a fivefold and fourfold decrease in sensitivity to sapatinib and lapatinib respectively. Taken together with the increased HO-1 in sapatinib-treated tumors from the MMTV-NIC-PTEN^+/−^ mice this indicates a role for HO-1 in driving sapatinib resistance. Interestingly the increased expression of HO-1 in the sapatinib-treated tumors did not result from increased activity of an NRF2-mediated oxidative stress response. NRF2-independent induction of HO-1 has been reported during keratinocyte differentiation and muscle atrophy [35–37]. During muscle atrophy the NRF2-independent expression of HO-1 is driven by the transcription factor FoxO1 [35]. However, as FoxO1 is activated by reactive oxygen species it is unlikely that the induction of HO-1 in the sapatinib-treated tumors is via FoxO1 and further work is required to delineate the pathways involved.

The mechanisms underlying resistance mediated by HO-1 involve induction of autophagy, response to oxidative stress or a combination of both [31, 32, 38–41]. Here we show in the context of HER2 inhibition that the cytoprotective effect of HO-1 is dependent on the induction of autophagy with inhibition of autophagy enhancing both sapatinib- and lapatinib-induced cell death. The increased autophagy evidenced in the sapatinib-treated tumors in the MMTV-NIC-PTEN^+/−^ mice provides further support for HO-1-induced autophagy as a potential mechanism of resistance to HER2-targeted therapies. Several signaling pathways have been implicated in the induction of autophagy by HO-1 including mTOR, ERK, JNK and p38 MAPK [33, 42, 43]. mTOR signallng plays an important role in the regulation of autophagy, however, we were not able to detect activation of mTOR signaling in either the SKBR3 empty vector or HO-1 expressing cell lines. This is in contrast to an increased activation of ERK and JNK in the SKBR3 HO-1 overexpressing cells. The relevance of this to sapatinib resistance is not clear as we saw no increase in ERK activation in the sapatinib-treated tumors. However, a more detailed analysis of the temporal changes involved with these signaling pathways during the development of resistance will be required to understand the pathways involved.

Nuclear translocation of HO-1 has been demonstrated following its proteolytic cleavage and this can result in increased proliferation and invasion independent of its enzymatic activity [44]. Furthermore, nuclear targeting of HO-1 was required for its cytoprotective effects against imatinib [19]. This may be manifested by the activation of transcriptional programs by nuclear HO-1 that protect against oxidative stress [45, 46]. However, the role of nuclear HO-1 in the control of autophagy is unknown.

Several studies have shown that molecular and pharmacological perturbation of HO-1 can enhance drug sensitivity [17, 18, 47, 48] raising the possibility that HO-1 may be a potential therapeutic target [15, 16]. This has led to significant investment in the design and synthesis of HO-1 inhibitors. Metalloporphyrins represent the first class of competitive HO-1 inhibitors that were developed: they compete with heme binding to HO-1 thereby reducing its activity. However, their clinical activity has been limited by off-target effects as they also interact with other heme-containing enzymes including nitric oxide synthase, soluble guanylate cyclase and cytochrome P450. Although considerable advances have been made to develop non-porphyrin inhibitors with more favorable bioavailability and increased selectivity for HO-1, there are limited reports of their anti-tumor activity in vivo with potency remaining an issue [49]. However, our data support the future development of more potent and selective HO-1 inhibitors, which may provide additional treatment options for HER2-positive treatment resistant disease. Additionally, treatment with autophagy inhibitors presents an alternative treatment option, although current autophagy inhibitors in clinical trials also suffer from off-target effects, restricting their use [50].

## Electronic supplementary material

Below is the link to the electronic supplementary material.

Supplementary material 1 (PDF 132 kb)
