# Supplementary material for: HO-1 drives autophagy as a mechanism of resistance against HER2-targeted therapies
Source: Breast Cancer Res Treat. 2019 Nov 8;179(3):543–55. doi: 10.1007/s10549-019-05489-1 (PMC6997276; doi:10.1007/s10549-019-05489-1)
Supplement: Supplementary file 1 — Supplementary material 1 (PDF 132 kb) [file 10549_2019_5489_MOESM1_ESM.pdf]

Breast Cancer Research & Treatment

Supplementary File: HO-1 drives autophagy as a mechanism of resistance against HER2-targeted therapies

Natasha Tracey[1], Helen Creedon[1], Alain J Kemp[1], Jayne Culley[1], Morwenna Muir[1], Teresa Klinowska[2], Valerie G Brunton[1*]

[1]Edinburgh Cancer Research UK Centre, Institute of Genetics & Molecular Medicine, University of Edinburgh, Crewe Road South, Edinburgh, EH4 2XR and [2]AstraZeneca, Oncology, IMED Biotech Unit, Cambridge, UK

[*]Correspondence: Professor Valerie Brunton

Email v.brunton@ed.ac.uk

Phone +44 131 650 8500

FAX +44 131 777 3520

Supplementary Figure 1

a

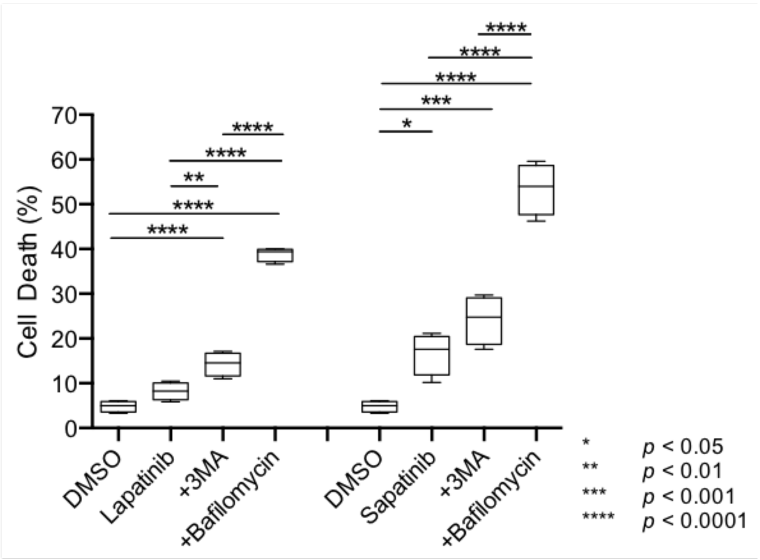

b

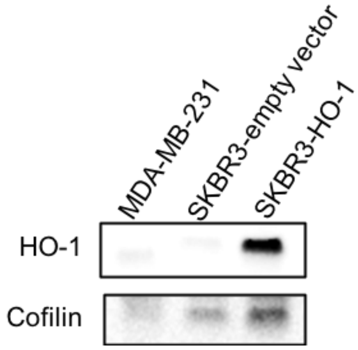

Supplementary Fig.ure 1 MDA-MB-231 cells were treated for 48 hours with dimethyl sulfoxide (DMSO; 0.01%), sapatinib (0.67 μM) or lapatinib (5 μM) in the presence or absence of autophagy inhibitors 3-methyladenine (3-MA; 5 mM) or bafilomycin A1 (bafilomycin; 5 nM). Cells were stained with propidium iodide and percentage of cell death was analysed using an Accuri™ C6 Flow Cytometer. Results presented as box and whisker plot, minimum of three biological repeats. All conditions were compared to DMSO control and single agent treatments. One-way ANOVA, Bonferroni's post-hoc test, not significant=NS, p<0.05=*, p<0.01=**, p<0.001=***. (b) Western blot analysis of HO-1 in SKBR3 vector, SKBR3-HO-1 and MDA-MB-231 cells. Cofilin was used as a loading control
